# Trabectedin is a promising antitumor agent potentially inducing melanocytic differentiation for clear cell sarcoma

**DOI:** 10.1002/cam4.1130

**Published:** 2017-07-26

**Authors:** Takaaki Nakai, Yoshinori Imura, Hironari Tamiya, Shutaro Yamada, Sho Nakai, Naohiro Yasuda, Keiko Kaneko, Hidetatsu Outani, Satoshi Takenaka, Kenichiro Hamada, Akira Myoui, Nobuhito Araki, Takafumi Ueda, Kazuyuki Itoh, Hideki Yoshikawa, Norifumi Naka

**Affiliations:** ^1^ Department of Orthopaedic Surgery Osaka University Graduate School of Medicine 2‐2 Yamadaoka Suita Osaka 565‐0871 Japan; ^2^ Musculoskeletal Oncology Service Osaka International Cancer Institute 3‐1‐69, Otemae Chuo‐ku Osaka 541‐8567 Japan; ^3^ Department of Orthopaedic Surgery Osaka National Hospital 2‐1‐14 Hoenzaka Chuo‐ku Osaka 540‐0006 Japan; ^4^ Research Institute Nozaki Tokushukai 2‐10‐50 Tanigawa Daito Osaka 574‐0074 Japan

**Keywords:** Clear cell sarcoma, differentiation therapy, melanocytic differentiation, trabectedin

## Abstract

Clear cell sarcoma is an aggressive soft tissue sarcoma and highly resistant to conventional chemotherapy and radiation therapy. This devastating disease is defined by EWSR1‐ATF1 fusion gene resulting from chromosomal translocation t(12;22)(q13;q12) and characterized by melanocytic differentiation. A marine‐derived antineoplastic agent, trabectedin, inhibits the growth of myxoid liposarcoma and Ewing sarcoma by causing adipogenic differentiation and neural differentiation, respectively. In this study, we examined the antitumor effects and mechanism of action of trabectedin on human clear cell sarcoma cell lines. We showed that trabectedin decreased the cell proliferation of five clear cell sarcoma cell lines in a dose‐dependent manner in vitro and reduced tumor growth of two mouse xenograft models. Flow cytometry and immunoblot analyses in vitro and immunohistochemical analysis in vivo revealed that trabectedin‐induced G2/M cell cycle arrest and apoptosis. Furthermore, trabectedin increased the expression of melanocytic differentiation markers along with downregulation of ERK activity in vitro and the rate of melanin‐positive cells in vivo. These results suggest that trabectedin has potent antitumor activity against clear cell sarcoma cells by inducing cell cycle arrest, apoptosis, and, in part, by promoting melanocytic differentiation through inactivation of ERK signaling. Our present study indicates that trabectedin is a promising differentiation‐inducing agent for clear cell sarcoma.

## Introduction

Clear cell sarcoma (CCS) is a rare but highly malignant soft tissue sarcoma that typically develops in the tendons and aponeuroses of children and young adults 1, 2. CCS is very resistant to conventional chemotherapy and radiation therapy. The high rate of local and distant recurrence results in 5‐year overall survival rates of 30–67% 3, 4, 5, 6, 7, 8, 9, 10. Thus, there is an urgent need for novel therapeutic approaches against CCS.

Cytogenetic analysis of CCS revealed the presence of translocation t(12;22)(q13;q12), resulting in a chimeric EWSR1‐ATF1 gene 11, 12, 13, 14. CCS was originally considered to be a melanoma of soft tissue origin and was called “malignant melanoma of soft parts.” From this point of view, CCS has been proposed to arise from a progenitor neural crest cell with the potential for melanocytic differentiation and melanin synthesis 15, 16, 17.

Trabectedin (ET‐743; Yondelis^®^) is a marine‐derived natural product originally isolated from the Caribbean Sea squirt, *Ecteinascidia turbinat*a and currently prepared synthetically 18. It has potent antitumor activity in vitro and in vivo against ovarian cancer, breast cancer, non‐small‐cell lung cancer, melanoma, and soft tissue sarcoma (STS) 19, 20, 21, 22, 23. Clinical trials revealed that trabectedin is particularly active in sarcomas bearing translocations, namely myxoid liposarcoma, Ewing sarcoma, and synovial sarcoma 24, 25. The mechanisms of action of trabectedin seem to be unique and still poorly understood. It has been reported that trabectedin interferes with several transcription factors, DNA‐binding proteins, and DNA repair pathways, likely differing from other DNA‐interacting agents 26. It has also been demonstrated that trabectedin shows antitumor activity by inducing adipogenic differentiation in liposarcoma 27 and neural differentiation in Ewing sarcoma 28. However, the activity and potential mode of action of trabectedin against CCS is still unknown.

In this study, we examined the antitumor effects of trabectedin on human CCS cell lines in vitro and in vivo. Furthermore, we investigated the mechanisms of action of trabectedin, focusing on the melanocytic differentiation pathway.

## Materials and Methods

### Cell culture

The human CCS cell lines Hewga‐CCS 29 and Senju‐CCS were established in our laboratory. MP‐CCS‐SY and KAS were kindly provided by Dr. Moritake (Miyazaki University, Miyazaki, Japan) 30 and Dr. Nakamura (Japanese Foundation for Cancer Research, Tokyo, Japan), respectively 31. SU‐CCS1 was purchased from American Type Culture Collection. We performed reverse transcription–PCR (RT–PCR) and assessed the type of EWSR1‐ATF1 chimeric transcripts in all CCS cell lines according to the EWSR1‐ATF1 fusion types proposed by Panagopoulos, et al. 11 (Data S1, Fig. S1). All the cell lines were cultured in Dulbecco's Modified Eagle Medium (Life Technologies, Carlsbad, CA, USA) containing 10% FBS (Sigma‐Aldrich, St. Louis, MO, USA) at 37°C with 5% CO_2_ and 100% humidity.

### Quantitative real‐time PCR analysis

Real‐time PCR was performed, using a Step One Plus Real‐Time PCR System (Life Technologies) and Fast SYBR Green Master Mix (Life Technologies), in which each cDNA sample was evaluated and expression values were normalized to GAPDH. PCR primers (forward and reverse, respectively) were as follows: GAPDH (5′‐TGCACCACCAACTGCTTAGC‐3′ and 5′‐ACTGTGGTCATGAGTCCTTCCA‐3′) and MITF (5′‐GAGGCAGTGGTTTGGGCTT‐3′ and 5′‐AATTCTGCACCCGGGAATC‐3′).

### Reagents and antibodies

Trabectedin was gifted by TAIHO Pharmaceutical, Co. LTD (Tsukuba, Japan). Methotrexate (MTX) hydrate and doxorubicin (DOX) were purchased by Sigma‐Aldrich (St. Louis, MO, USA) and Wako Pure Chemical Industries (Osaka, Japan), respectively. The ERK inhibitor, SCH772984, was purchased from Cayman Chemical (Ann Arbor, MI, USA). The drugs were prepared in dimethyl sulfoxide (DMSO) and stored at −20°C. For in vitro examinations, the drugs were diluted in DMEM to the desired concentration. For in vivo experiments, trabectedin was further diluted with phosphate buffer pH 4.0 immediately before administration.

The following primary antibodies were used: anti‐cleaved caspase‐3 and anti‐beta‐actin (Cell Signaling Technology, Inc., Danvers, MA, USA); anti‐microphthalmia‐associated transcription factor (MITF) and anti‐activating transcription factor 1 (ATF1) (Abcam, Cambridge, UK); anti‐PCNA, anti‐tyrosinase (TYR), and anti‐tyrosinase‐related protein 2 (TRP2) (Santa Cruz Biotechnology, Inc., Dallas, TX, USA). Horseradish peroxidase (HRP)‐conjugated secondary antibody was obtained from GE Healthcare Life Sciences (Pittsburg, PA, USA).

### Immunoblot analysis

For the lysate preparation, cells were first washed with phosphate‐buffered saline and lysed in radioimmunoprecipitation assay (RIPA) buffer (Thermo Scientific, Waltham, MA, USA). Protein concentrations were measured using the bicinchoninic acid method (Thermo Scientific). The cell lysates were separated on 4–12% Bis‐Tris gels (Life Technologies) and transferred to polyvinylidene difluoride membranes (Nippon Genetics, Tokyo, Japan). The membranes were incubated in 5% skim milk in Tris‐buffered saline with Tween 20 (TBS‐T) at room temperature. Blocked membranes were incubated with primary antibodies at 4°C overnight, followed by incubation with secondary antibodies at room temperature for 1 h. After washing in TBS‐T, immunoreactive bands were visualized using enhanced chemiluminescence (GE Healthcare Life Sciences).

### Cell proliferation assay

CCS cells were plated into 96‐well plates at a density of 2 × 10^3^ and incubated with trabectedin or the vehicle for 72 h. Cell proliferation rate was measured using the premix WST‐1 cell proliferation assay system (Takara Bio, Inc., Otsu, Japan). Absorbance was measured with a microplate reader and relative cell proliferation rate was calculated.

### Flow cytometry

5 × 10^5^ CCS cells per dish were plated in 10 cm culture dishes and grown overnight, followed by treatment with trabectedin or vehicle solution for 48 h. The cells were harvested and stained with propidium iodide (PI) solution (25 *μ*g/mL PI, 0.03% NP‐40, 0.02 mg/mL RNase A, 0.1% sodium citrate) for 30 min at room temperature. For cell cycle analysis, we used a BD FACSCanto II flow cytometer (Becton Dickinson (BD) Biosciences, San Jose, CA, USA).

### In vivo mouse xenograft model

Five‐week‐old male BALB/c nu/nu mice (SLC, Shizuoka, Japan) were housed at the Institute of Experimental Animal Sciences, Osaka University Medical School, in accordance with a guideline approved by the Institutional Animal Care and Use Committee of the Osaka University Graduate School of Medicine. For the xenograft tumor growth assay, 1 × 10^7^ CCS cells were injected subcutaneously into the left side of the back. Tumor volumes were measured twice per week with calipers and calculated by the formula (A × B^2^)/2, where A was the longest diameter and B was the shortest diameter of the tumor. When the average diameter of tumors reached 5 mm, therapy was initiated. The mice were randomized and divided into trabectedin‐treated or vehicle‐treated groups. Trabectedin was intravenously injected through the tail vein at the dose of 0.15 mg/kg, every 7 days for three cycles. After 18 days of treatment, all mice were euthanized and the tumor weight was measured. The tumors were resected for histological analysis. Body weight of each mouse was measured once a week for the evaluation of side effects.

### Histological analysis

Specimens of xenograft tumors were fixed in 10% neutral‐buffered formalin, embedded in paraffin, and sectioned in 4 *μ*m thicknesses. Paraffin‐embedded sections were deparaffinized and dehydrated. After antigen retrieval (boiled at 95°C for 20 min in a 10 mmol/L citrate buffer), endogenous peroxidase activity was blocked for 10 min with methanol containing 3% H_2_O_2_. The sections were reacted for 1 h with TBS containing 2% bovine serum albumin at room temperature. The sections were incubated with designated antibodies at 4°C overnight, followed by 1 h incubation with secondary antibodies and stained with 3,3′‐diaminobenzidine tetrahydrochloride (Dako, Glostrup, Denmark). Finally, the sections were counterstained using hematoxylin. To detect melanin, paraffin sections were stained using Fontana–Masson stain as described previously 32.

### Statistical analysis

All data are expressed as means ± SDs. We used Student's t‐test for biological assays and Mann–Whitney's U test for animal experiments to determine significant differences. Values of *P* < 0.05 were considered significant.

## Results

### Trabectedin suppressed the growth of CCS cell lines in vitro

**Figure 1:**
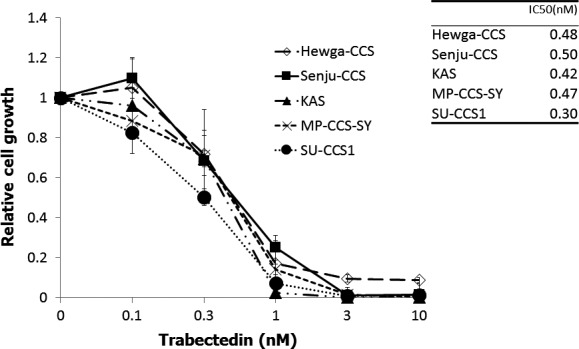
Trabectedin inhibits CCS cell growth in vitro. The CCS cells were incubated with various concentrations of trabectedin for 72 h. Cell proliferation was determined by WST‐1 assay. The IC_50_ values were calculated and shown in the table. Bars: SD.

Cell proliferation assays were performed to examine the antitumor activity of trabectedin against 5 CCS cell lines in vitro. Trabectedin suppressed proliferation of all CCS cell lines in a dose‐dependent manner (Fig. 1). The 50% inhibitory concentration (IC_50_) values of trabectedin were as follows: Hewga‐CCS: 0.48 nmol/L, Senju‐CCS: 0.87 nmol/L, SU‐CCS1: 0.30 nmol/L, MP‐CCS‐SY: 0.47 nmol/L, KAS: 0.42 nmol/L (Fig. 1).

### Trabectedin induced G2/M cell‐cycle arrest and apoptosis in CCS cell lines

**Figure 2:**
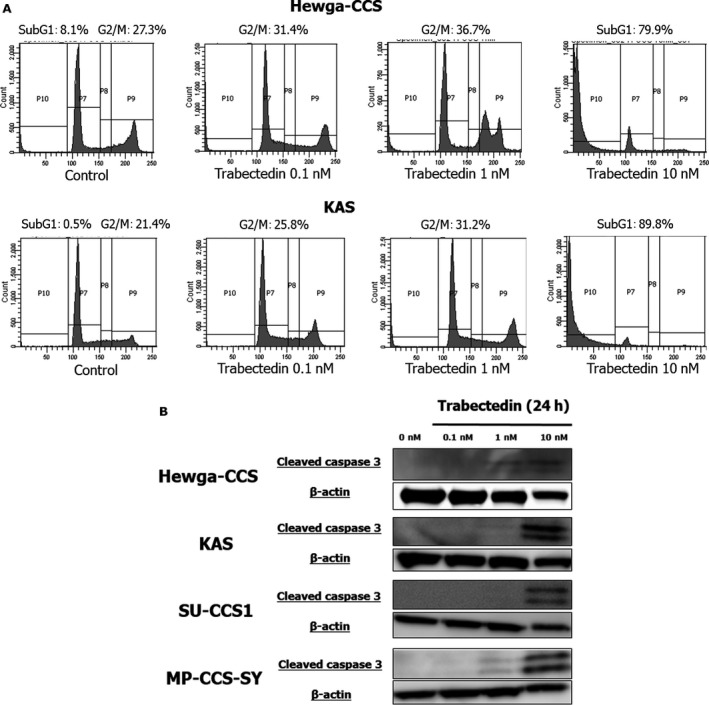
Trabectedin induces G2/M cell‐cycle arrest and apoptosis in CCS cells. (A) Hewga‐CCS and KAS cells were exposed to 0.1–10 nmol/L trabectedin or vehicle for 48 h. After exposure, cells were stained with PI and analyzed by flow cytometry. (B) After treatment of trabectedin or vehicle in CCS cells, the protein expressions were observed by Immunoblot analyses.

Flow cytometry analyses showed that 1 nmol/L trabectedin induced G2/M cell‐cycle arrest, and 10 nmol/L trabectedin increased the number of cells in sub‐G1 phase in Hewga‐CCS and KAS cells (Fig. 2A). Furthermore, cleavage of caspase‐3 was enhanced dose‐dependently after trabectedin exposure by immunoblot (Fig. 2B). These findings indicated that trabectedin inhibited the cell proliferation in all CCS cell lines by inducing G2/M cell cycle arrest and apoptosis in vitro.

### The expression of melanocytic differentiation markers was upregulated by treatment with trabectedin in vitro

**Figure 3:**
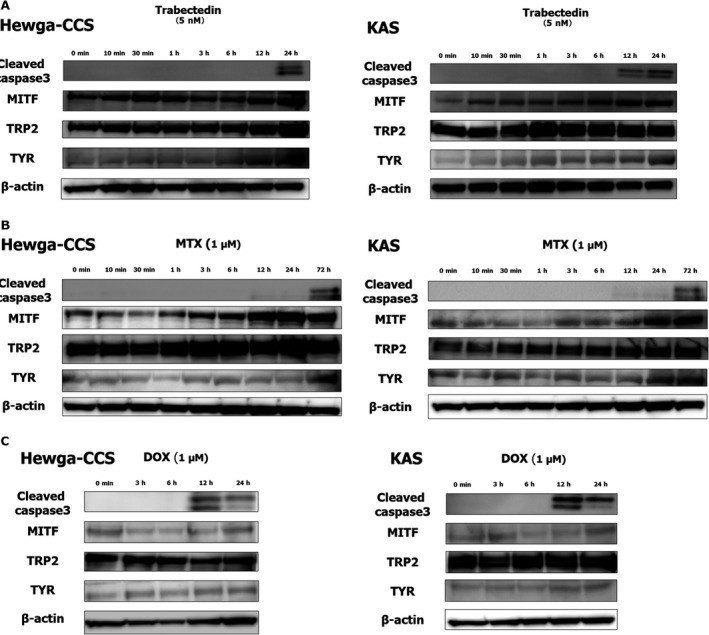
Melanocytic differentiation markers in Hewga‐CCS and KAS were upregulated by the treatment of trabectedin and MTX, but not DOX. Hewga‐CCS and KAS were treated with (A) 5 nmol/L trabectedin for 0–24 h, (B) 1 uM MTX for 0–72 h and (C) 1 uM DOX for 0–24 h. The protein expressions were evaluated by Immunoblot analyses.

We examined the expression of melanocytic differentiation markers such as MITF, TYR, and TRP2 in Hewga‐CCS and KAS cells treated with trabectedin, MTX, and DOX by immunoblot. It was reported that MTX increased MITF expression and induced melanocytic differentiation in melanoma cells 33. DOX is a conventional chemotherapeutic drug and was used as a control. All three drugs induced cleavage of caspase‐3 in both cell lines (Fig. 3). Interestingly, prior to cleavage of caspase‐3, expression of melanocytic differentiation markers was upregulated by treatment with trabectedin and MTX (Fig. 3B). Treatment with DOX did not upregulate these markers (Fig. 3C). These results suggested that trabectedin might inhibit the growth of CCS cells by inducing melanocytic differentiation and apoptosis.

### Trabectedin caused inactivation of ERK and increased MITF protein level

**Figure 4:**
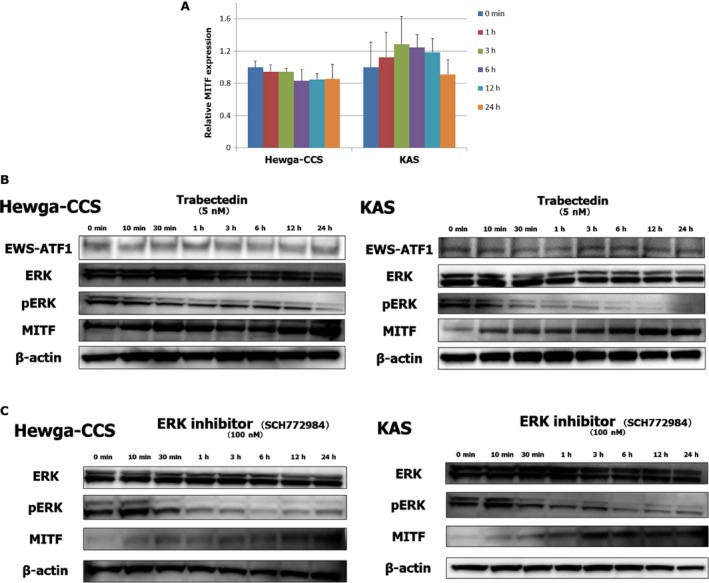
Trabectedin did not enhance the mRNA expression of MITF. Moreover, the drug did not affect the expression of EWSR1‐ATF1 fusion protein. Both trabectedin and a selective ERK inhibitor, SCH772984, decreased the ERK signaling and increased the protein level of MITF. Hewga‐CCS and KAS were treated with 5 nmol/L trabectedin or 100 nmol/L SCH772984 for 0–24 h. (A) Total RNA was extracted, and MITF transcription was quantified by qRT‐PCR. Values mean ± SD. (B, C) The protein expressions were assessed by Immunoblot analyses.

To elucidate the reason why trabectedin increased MITF protein level, we examined whether the drug induced MITF gene transcription. Unexpectedly, trabectedin did not upregulate the mRNA level of MITF in Hewga‐CCS and KAS cells (Fig. 4A). Further, the expression of the EWSR1‐ATF1 fusion protein was not affected by the treatment of trabectedin in both cells (Fig 4B). These results suggested that the upregulation of MITF protein levels by trabectedin treatment was neither attributable to the transcriptional activation of MITF nor mediated by the interaction between trabectedin and EWSR1‐ATF1.

Previous studies showed that the activation of ERK was followed by MITF ubiquitination and degradation 34, 35, thus we evaluated the phosphorylation status of ERK in response to trabectedin treatment. Consistent with those studies, the reduction in ERK phosphorylation via trabectedin exposure was accompanied by the upregulation of MITF (Fig. 4B). Since trabectedin appeared to induce melanocytic differentiation by deregulating the activation of ERK, we investigated whether its similar effect on CCS was promoted by the treatment of SCH772984, a specific inhibitor of ERK signaling. Similar to trabectedin, SCH772984 suppressed ERK activation and upregulated MITF protein level in Hewga‐CCS and KAS cells (Fig. 4C). These findings suggested that the inhibition of the ERK signaling might play a pivotal role in trabectedin‐induced melanocytic differentiation.

### Trabectedin suppressed the growth of CCS xenograft tumors

**Figure 5:**
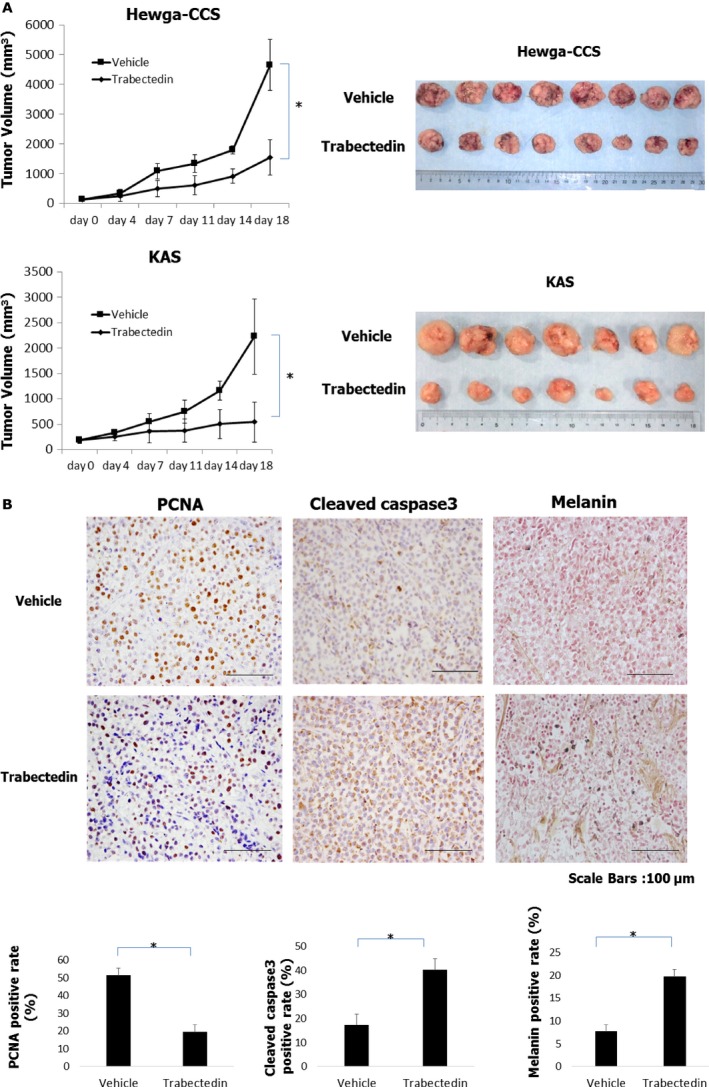
Trabectedin abrogated the growth of Hewga‐CCS and KAS xenograft tumors. (A) Hewga‐CCS (*n* = 8/group each) and KAS (*n* = 7/group each) xenograft tumors were treated with 0.15 mg/kg trabectedin intravenously injected. (B) The rate of PCNA‐positive tumor cells, cleaved caspase‐3‐positive cells, and melanin‐positive cells were counted in Hewga‐CCS xenograft tumors. *P < 0.01.

We evaluated the antitumor effects of trabectedin against Hewga‐CCS and KAS xenograft tumors. Trabectedin at a dose of 0.15 mg/kg was intravenously injected once every 7 days for three cycles. Administration of trabectedin notably suppressed the growth of CCS xenograft tumors compared to the vehicle control (Fig. 5A). Body weight loss of the mice was not observed and drug treatment was well‐tolerated with no toxicity in this study. Consistent with the in vitro data, a decrease in the rate of PCNA‐positive tumor cells and an increase in that of cleaved caspase‐3‐positive cells and melanin‐positive cells were observed in trabectedin‐treated CCS xenograft tumors (Fig. 5B). These data suggested that trabectedin showed antineoplastic activity against CCS cells, and supported the hypothesis that one of the mechanisms of action of trabectedin might be associated with promotion of melanocytic differentiation in addition to the induction of cell cycle arrest and apoptosis.

## Discussion

Prior publications suggest that maintaining an undifferentiated state is a crucial aspect of cancer tumorigenesis since cancer cells display suspended differentiation properties compared to normal cells 36, 37. Extended from those studies, several investigators presented an idea that the lineage differentiation block associated with both fusion protein expression and sarcoma‐associated genetic abnormalities played a critical role in translocation‐based sarcomagenesis 38, 39, 40. For instance, Tirode, et al. demonstrated that EWSR1‐FLI1 fusion protein blocked terminal differentiation of mesenchymal stem cells, leading to Ewing sarcoma tumorigenesis 38. Similarly, Charytonowicz, et al. reported that PAX3/PAX7‐FOXO1 translocation promoted the differentiation arrest in the myogenic lineage, resulting in alveolar rhabdomyosarcoma sarcomagenesis 39. These results suggest that suspension of undifferentiated state is required for tumorigenic process and targeting impaired terminal differentiation could be a novel therapeutic strategy for translocation‐based sarcomas.

Differentiation therapies aim to force the cancer cell to resume the process of maturation and tend to have less toxicity than conventional cancer treatments 41. Many approaches for differentiation therapy in cancer using histone deacetylase inhibitors 42, retinoids 43, or peroxisome proliferator‐activated receptors *γ* agonists 44 exhibited encouraging results in both in vitro and in vivo experiments. Among them, the highly successful clinical application of differentiation therapy was all‐trans‐retinoic acid‐based therapy against acute promyelocytic leukemia 45.

CCS is characterized by a chromosomal translocation, t(12;22)(q13;q12), that leads to the fusion of EWSR1 gene with a CREB‐family transcription factor gene (ATF1, or more rarely CREB1) 11, 12, 13, 14. These translocations provided a means of defining CCS and distinguishing it from malignant melanoma. Recent studies showed that CCS was a neural crest‐derived malignancy as malignant melanoma 15, 16, 17. Several lines of evidence indicated that trabectedin was more effective against translocation‐related sarcoma, such as myxoid liposarcoma, Ewing sarcoma and synovial sarcoma 24, 25. It was hypothesized that the greater sensitivity of these sarcomas was related to the ability of trabectedin to interact with the fusion gene product. Beyond expectations, we found that trabectedin did not influence the expression of EWSR1‐ATF1 protein.

This study demonstrated that treatment of CCS with trabectedin suppressed cell proliferation, increased the number of cells in G2/M and sub‐G1 phase in vitro, and induced cleavage of caspase‐3 both in vitro and in vivo. Previous studies revealed that trabectedin exerted differentiation inducing and antitumor effects for a subset of translocation‐related sarcomas, including myxoid liposarcoma 27 and Ewing sarcoma 28. In this study, we also noticed that trabectedin treatment upregulated the expression of melanocytic differentiation markers including MITF in vitro and melanin‐positive cells in vivo, even though it did not enhance the transcriptional activity for MITF. Intriguingly, recent works suggested that MITF protein levels were regulated by ERK‐induced ubiquitination and degradation in melanoma cells 34, 35. In agreement with this, we noted that the reduction in ERK signaling was associated with the increase in MITF protein levels by the treatment with trabectedin or an ERK inhibitor. These findings suggested that trabectedin might induce melanocytic differentiation on CCS as a result of the reduction in ERK activity, aside from the interaction with the EWSR1‐ATF1 fusion gene product.

Our findings strongly indicate that trabectedin exerts antitumor effects via induction of G2/M cell cycle arrest, apoptosis, and, in part, the acceleration of melanocytic differentiation against CCS cell lines. Taken together, we conclude that trabectedin should be a promising therapeutic option and might be a novel differentiation therapy agent for CCS.

## Conflict of Interest

We declare no conflicts of interest.

## Supporting information


**Data S1.** EWSR1‐ATF1 cDNA was identified by PCR. PCR primers were as follows: EWSR1 forward primer 5′‐TCCTACAGCCAAGCTCCAAGTC‐3′ and ATF1 reverse primer 5′‐ACTCGGTTTTCCAGGCATTTCAC‐3′.

**Figure S1.** RT–PCR with EWSR1 forward primer and ATF1 reverse primer that amplifies a 779‐base pair (bp) PCR product in the type 1 EWSR1‐ATF1 transcript, a 464‐bp PCR product in type 2, and a 713‐bp PCR product in type 3. The type 1 transcript of EWSR1‐ATF1 was detected in Senju‐CCS, SU‐CCS1, and MP‐CCS‐SY cells. The type 2 and the type 3 transcripts of EWSR1‐ATF1 were detected in Hewga‐CCS and KAS cells, respectively.

## References

[1] Enzinger, F. M. 1965 Clear‐cell sarcoma of tendons and aponeuroses: an analysis of 21 cases. Cancer 18:1163–1174. 14332545 10.1002/1097-0142(196509)18:9<1163::aid-cncr2820180916>3.0.co;2-0

[2] Goldblum, J. R. , A. L. Flope , and S. W. Weiss . 2013Enzinger & Weiss's Soft Tissue Tumors. 6th edn. Pp. 886–894. Soft Tissue Tumors Showing Melanocytic Differentiation. Elsevier, Philadelphia.

[3] Sara, A. S. , H. I. Evans , and R. S. Benjamin . 1990 Malignant melanoma of soft parts (clear cell sarcoma): a study of 17 cases, with emphasis on prognostic factors. Cancer 65:367–374. 2295060 10.1002/1097-0142(19900115)65:2<367::aid-cncr2820650232>3.0.co;2-x

[4] el‐Naggar, A. K. , N. G. Ordonez , A. Sara , D. McLemore , and J. G. Batsakis . 1991 Clear cell sarcomas and metastatic soft tissue melanomas. Cancer 67:2173–2179. 2004337 10.1002/1097-0142(19910415)67:8<2173::aid-cncr2820670828>3.0.co;2-o

[5] Lucas, D. R. , A. G. Nascimento , and F. H. Sim . 1992 Clear cell sarcoma of soft tissues: mayo Clinic experience with 35 cases. Am. J. Surg. Pathol. 16:1197–1204. 1463095 10.1097/00000478-199212000-00006

[6] Montgomery, E. A. , J. M. Meis , A. G. Ramos , D. M. Frisman , and K. L. Martz . 1993 Clear cell sarcoma of tendons and aponuroses: a clinicopathologic study of 58 cases with analysis of prognostic factors. Int. J. Surg. Pathol. 1:89–100.

[7] Deenik, W. , W. J. Mooi , E. J. Rutgers , J. L. Peterse , A. A. Hart , and B. B. Kroon . 1999 Clear cell sarcoma (malignant melanoma) of soft parts: a clinicopathologic study of 30 cases. Cancer 86:969–975. 10491522

[8] Finley, J. W. , B. Hanypsiak , B. Mcgrath , W. Kraybill , and J. F. Gibbs . 2001 Clear cell sarcoma: the Roswell Park experience. J. Surg. Oncol. 77:16–20. 11344475 10.1002/jso.1057

[9] Ferrari, A. , M. Casanova , G. Bisogno , A. Mattke , C. Meazza , L. Gandola , et al. 2002 Clear cell sarcoma of tendons and aponeuroses in pediatric patients: a report from the Italian and German Soft Tissue Sarcoma Cooperative Group. Cancer 94:3269–3276. 12115360 10.1002/cncr.10597

[10] Takahira, T. , Y. Oda , S. Tamiya , H. Yamamoto , K. Kawaguchi , C. Kobayashi , et al. 2004 Alteration of the p16INK4a/p14ARF pathway in clear cell sarcoma. Cancer Sci. 95:651–655. 15298727 10.1111/j.1349-7006.2004.tb03324.x PMC11158930

[11] Panagopoulos, I. , F. Mertens , M. Debiec‐Rychter , M. Isaksson , J. Limon , I. Kardas , et al. 2002 Molecular genetic characterization of the EWS/ATF1 fusion gene in clear cell sarcoma of tendons and aponeuroses. Int. J. Cancer 99:560–567. 11992546 10.1002/ijc.10404

[12] Bridge, J. A. , C. Sreekantaiah , J. R. Neff , and A. A. Sandberg . 1991 Cytogenetic findings in clear cell sarcoma of tendons and aponeuroses. Malignant melanoma of soft parts. Cancer Genet. Cytogenet. 52:101–106. 2009504 10.1016/0165-4608(91)90059-4

[13] Stenman, G. , L. G. Kindblom , and L. Angervall . 1992 Reciprocal translocation t(12;22)(q13;q13) in clear‐cell sarcoma of tendons and aponeuroses. Gene Chromosome Canc. 4:122–127. 10.1002/gcc.2870040204 1373311

[14] Zucman, J. , O. Delattre , C. Desmaze , A. L. Epstein , G. Stenman , F. Speleman , et al. 1993 EWS and ATF‐1 gene fusion induced by t(12:22) translocation in malignant melanoma of soft parts. Nat. Genet. 4:341–345. 8401579 10.1038/ng0893-341

[15] Segal, N. H. , P. Pavlidis , W. S. Noble , C. R. Antonescu , A. Viale , U. V. Wesley , et al. 2003 Classification of clear‐cell sarcoma as a subtype of melanoma by genomic profiling. J. Clin. Oncol. 21:1775–1781. 12721254 10.1200/JCO.2003.10.108

[16] Straessler, K. M. , K. B. Jones , H. Hu , H. Jin , M. van de Rijn , M. R. Capecchi , et al. 2013 Modeling clear cell sarcomagenesis in the mouse: cell of origin differentiation state impacts tumor characteristics. Cancer Cell 23:215–227. 23410975 10.1016/j.ccr.2012.12.019 PMC3640275

[17] Yamada, K. , T. Ohno , H. Aoki , K. Semi , A. Watanabe , H. Moritake , et al. 2013 EWS/ATF1 expression induces sarcomas from neural crest‐derived cells in mice. J. Clin. Invest. 123:600–610. 23281395 10.1172/JCI63572 PMC3561811

[18] Cuevas, C. , and A. Francesch . 2009 Development of Yondelis (trabectedin, ET‐743). A semisynthetic process solves the supply problem. Nat. Prod. Rep. 26:322–337. 19240944 10.1039/b808331m

[19] Valoti, G. , M. I. Nicoletti , A. Pellegrino , J. Jimeno , H. Hendriks , M. D'Incalci , et al. 1998 Ecteinascidin‐743, a new marine natural product with potent antitumor activity on human ovarian carcinoma xenografts. Clin. Cancer Res. 4:1977–1983. 9717828

[20] Hendriks, H. R. , H. H. Fiebig , R. Giavazzi , S. P. Langdon , J. M. Jimeno , and G. T. Faircloth . 1999 High antitumour activity of ET743 against human tumor xenografts from melanoma, non‐small‐cell lung and ovarian cancer. Ann. Oncol. 10:1233–1240. 10586342 10.1023/a:1008364727071

[21] Li, W. W. , N. Takahashi , S. Jhanwar , C. Cordon‐Cardo , Y. Elisseyeff , J. Jimeno , et al. 2001 Sensitivity of soft tissue sarcoma cell lines to chemotherapeutic agents: identification of ecteinascidin‐743 as a potent cytotoxic agent. Clin. Cancer Res. 7:2908–2911. 11555609

[22] Fayette, J. , I. R. Coquard , L. Alberti , H. Boyle , P. Méeus , A. V. Decouvelaere , et al. 2006 ET‐743: a novel agent with activity in soft‐tissue sarcomas. Curr. Opin. Oncol. 18:347–353. 16721129 10.1097/01.cco.0000228740.70379.3f

[23] Yasui, H. , Y. Imura , Hamada K. Outani , T. Nakai , S. Yamada , et al. 2016 Trabectedin is a promising antitumour agent for synovial sarcoma. J. Chemother. 28:417–424. 27077926 10.1080/1120009X.2015.1133013

[24] Blay, J. Y. , M. G. Leahy , B. B. Nguyen , S. R. Patel , P. Hohenberger , A. Santoro , et al. 2014 Randomised phase III trial of trabectedin versus doxorubicin‐based chemotherapy as first‐line therapy in translocation‐related sarcomas. Eur. J. Cancer 50:1137–1147. 24512981 10.1016/j.ejca.2014.01.012

[25] Martin‐Broto, J. , A. L. Pousa , Penas R. de Las , X. García Del Muro , A. Gutierrez , J. Martinez‐Trufero , et al. 2016 Randomized Phase II Study of Trabectedin and Doxorubicin Compared With Doxorubicin Alone as First‐Line Treatment in Patients With Advanced Soft Tissue Sarcomas: a Spanish Group for Research on Sarcoma Study. J. Clin. Oncol. 34:2294–2302. 27185843 10.1200/JCO.2015.65.3329

[26] D'Incalci, M. , and C. M. Galmarini . 2010 A review of Trabectedin (ET‐743): a Unique Mechanism of Action. Mol. Cancer Ther. 9:2157–2163. 20647340 10.1158/1535-7163.MCT-10-0263

[27] Forni, C. , M. Minuzzo , E. Virdis , E. Tamborini , M. Simone , M. Tavecchio , et al. 2009 Trabectedin (ET‐743) promotes differentiation in myxoid liposarcoma tumors. Mol. Cancer Ther. 8:449–457. 19190116 10.1158/1535-7163.MCT-08-0848

[28] Amaral, A. T. , C. Garofalo , R. Frapolli , M. C. Manara , C. Mancarella , S. Uboldi , et al. 2015 Trabectedin efficacy in Ewing sarcoma is greatly increased by combination with anti‐IGF signaling agents. Clin. Cancer Res. 21:1373–1382. 25609059 10.1158/1078-0432.CCR-14-1688

[29] Outani, H. , T. Tanaka , T. Wakamatsu , Y. Imura , K. Hamada , N. Araki , et al. 2014 Establishment of a novel clear cell sarcoma cell line (Hewga‐CCS), and investigation of the antitumor effects of pazopanib on Hewga‐CCS. BMC Cancer 14:455. 24946937 10.1186/1471-2407-14-455 PMC4076438

[30] Moritake, H. , T. Sugimoto , Y. Asada , M. A. Yoshida , Y. Maehara , A. L. Epstein , et al. 2002 Newly established clear cell sarcoma (malignant melanoma of soft parts) cell line expressing melanoma‐associated Melan‐A antigen and overexpressing C‐MYC oncogene. Cancer Genet. Cytogenet. 135:48–56. 12072203 10.1016/s0165-4608(01)00641-0

[31] Jishage, M. , T. Fujino , Y. Yamazaki , H. Kuroda , and T. Nakamura . 2003 Identification of target genes for EWS/ATF‐1 chimeric transcription factor. Oncogene 22:41–49. 12527906 10.1038/sj.onc.1206074

[32] Kwon‐Chung, K. J. , W. B. Hill , and E. Bennett . 1981 New, special stain for histopathological diagnosis of cryptococcosis. J. Clin. Microbiol. 13:383–387. 6162857 10.1128/jcm.13.2.383-387.1981 PMC273793

[33] Saez‐Ayala, M. , M. F. Montenegro , L. Sanchez‐Del‐Campo , M. P. Fernández‐Pérez , S. Chazarra , R. Freter , et al. 2013 Directed Phenotype Switching as an Effective Antimelanoma Strategy. Cancer Cell 24:105–119. 23792190 10.1016/j.ccr.2013.05.009

[34] Wu, M. , T. J. Hemesath , C. Takemoto , M. A. Horstmann , A. G. Wells , E. R. Price , et al. 2000 c‐Kit triggers dual phosphorylations, which couple activation and degradation of the essential melanocyte factor Mi. Genes Dev. 14:301–312. 10673502 PMC316361

[35] Kim, M. S. , S. H. Bang , J. H. Kim , H. J. Shin , J. H. Choi , and S. E. Chang . 2015 Tranexamic Acid Diminishes Laser‐Induced Melanogenesis. Ann. Dermatol. 27:250–256. 26082580 10.5021/ad.2015.27.3.250 PMC4466276

[36] Harris, H. 2005 A long view of fashions in cancer research. BioEssays 27:833–838. 16015588 10.1002/bies.20263

[37] Shah, N. , and S. Sukumar . 2010 The Hox genes and their roles in oncogenesis. Nat. Rev. Cancer 10:361–370. 20357775 10.1038/nrc2826

[38] Tirode, F. , K. Laud‐Duval , A. Prieur , B. Delorme , P. Charbord , and O. Delattre . 2007 Mesenchymal stem cell features of Ewing tumors. Cancer Cell 11:421–429. 17482132 10.1016/j.ccr.2007.02.027

[39] Charytonowicz, E. , I. Matushansky , J. D. Doménech , M. Castillo‐Martín , M. Ladanyi , C. Cordon‐Cardo , et al. 2012 PAX7‐FKHR fusion gene inhibits myogenic differentiation via NF‐kappaB upregulation. Clin. Transl. Oncol. 14:197–206. 22374423 10.1007/s12094-012-0784-4

[40] Haldar, M. , J. D. Hancock , C. M. Coffin , S. L. Lessnick , and M. R. Capecchi . 2007 A Conditional Mouse Model of Synovial Sarcoma: insights into a Myogenic Origin. Cancer Cell 11:375–388. 17418413 10.1016/j.ccr.2007.01.016

[41] Cruz, F. D. , and I. Matushansky . 2012 Solid Tumor Differentiation Therapy–Is It Possible? Oncotarget 3:559–567. 22643847 10.18632/oncotarget.512 PMC3388185

[42] Mai, A. , S. Massa , D. Rotili , I. Cerbara , S. Valente , R. Pezzi , et al. 2005 Histone deacetylation in epigenetics: an attractive target for anticancer therapy. Med. Res. Rev. 25:261–309. 15717297 10.1002/med.20024

[43] Garattini, E. , M. Gianni , and M. Terao . 2007 Cytodifferentiation by retinoids, a novel therapeutic option in oncology: rational combinations with other therapeutic agents. Vitam. Horm. 75:301–354. 17368321 10.1016/S0083-6729(06)75012-9

[44] Sertznig, P. , M. Seifert , W. Tilgen , and J. Reichrath . 2007 Present concepts and future outlook: function of peroxisome proliferator‐activated receptors (PPARs) for pathogenesis, progression, and therapy of cancer. J. Cell. Physiol. 212:1–12. 17443682 10.1002/jcp.20998

[45] Huang, M. E. , Y. C. Ye , S. R. Chen , J. R. Chai , J. X. Lu , L. Zhoa , et al. 1988 Use of all‐trans retinoic acid in the treatment of acute promyelocytic leukemia. Blood 72:567–572. 3165295

