# Supplementary figures and images for: Trabectedin is a promising antitumor agent potentially inducing melanocytic differentiation for clear cell sarcoma
Source: Cancer Med. 2017 Jul 26;6(9):2121–30. doi: 10.1002/cam4.1130 (PMC5603837; doi:10.1002/cam4.1130)

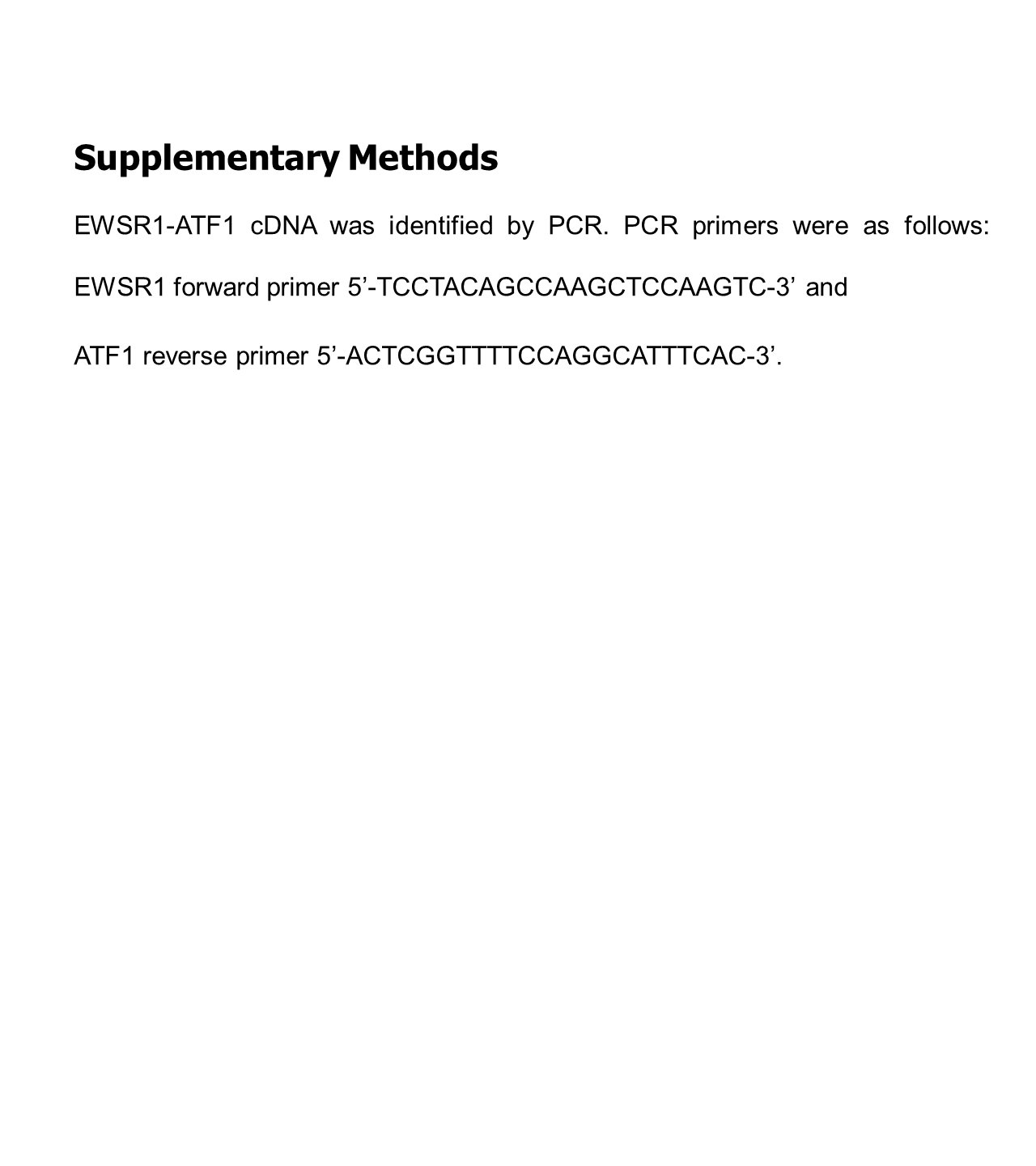

Supplement: Supplementary file 1 — Data S1. EWSR1‐ATF1 cDNA was identified by PCR. PCR primers were as follows: EWSR1 forward primer 5′‐TCCTACAGCCAAGCTCCAAGTC‐3′ and ATF1 reverse primer 5′‐ACTCGGTTTTCCAGGCATTTCAC‐3′. [file CAM4-6-2121-s001.tif]

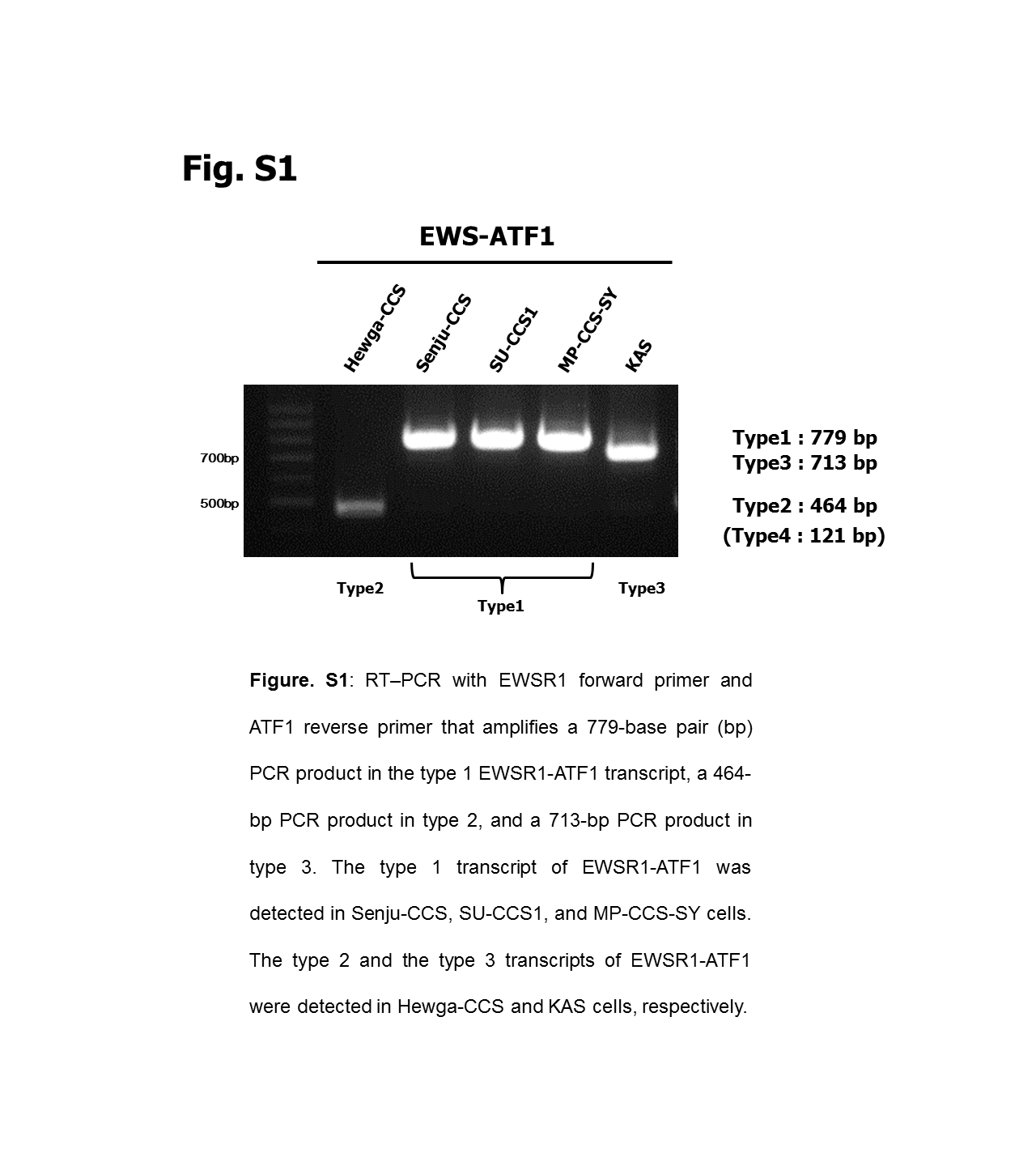

Supplement: Supplementary file 2 — Figure S1. RT–PCR with EWSR1 forward primer and ATF1 reverse primer that amplifies a 779‐base pair (bp) PCR product in the type 1 EWSR1‐ATF1 transcript, a 464‐bp PCR product in type 2, and a 713‐bp PCR product in type 3. The type 1 transcript of EWSR1‐ATF1 was detected in Senju‐CCS, SU‐CCS1, and MP‐CCS‐SY cells. The type 2 and the type 3 transcripts of EWSR1‐ATF1 were detected in Hewga‐CCS and KAS cells, respectively. [file CAM4-6-2121-s002.tif]
